# Investigation of the Prevalence of the HLA‐B*15:02 Allele in the Asian Populations: A Comprehensive Analysis Through Using AFND

**DOI:** 10.1002/hsr2.72903

**Published:** 2026-07-28

**Authors:** Mohitosh Biswas, Murshadul Alam Murad, Maliheh Ershadian, Chonlaphat Sukasem

**Affiliations:** ^1^ Department of Pharmacy, Faculty of Science University of Rajshahi Rajshahi Bangladesh; ^2^ Division of Pharmacogenomics and Personalized Medicine, Department of Pathology, Faculty of Medicine Ramathibodi Hospital Mahidol University Bangkok Thailand; ^3^ Laboratory for Pharmacogenomics, Somdech Phra Debaratana Medical Center (SDMC) Ramathibodi Hospital Bangkok Thailand; ^4^ Department of Pharmacy, School of Biomedical and Life Science Khwaja Yunus Ali University Sirajganj Bangladesh; ^5^ Pharmacogenomics and Precision Medicine, The Preventive Genomics & Family Check‐up Services Center, Bumrungrad International Hospital Bangkok Thailand

**Keywords:** clinical practice, *HLA*, HLA‐B*15:02, pharmacogenomics, severe cutaneous adverse drug reactions

## Abstract

**Background and Aims:**

The HLA‐B*15:02 allele is related to a high risk of severe cutaneous adverse drug reactions (SCARs) in patients taking certain antiepileptic drugs. However, its prevalence in the Asian population and the number of drugs linked to SCARs due to the carriage of this variant are relatively underexplored. This study sought to address these knowledge gaps.

**Methods:**

The prevalence of the HLA‐B*15:02 allele in Asian populations was estimated by using the data from the Allele Frequency Net Database. Weighted means, standard deviations, and 95% Confidence Interval (CI) were estimated for the study populations, and the chi‐square test was used to assess the statistical significance of variability in allele frequencies across populations. Drugs associated with SCARs due to HLA‐B*15:02 were identified from the clinical guidelines of the international pharmacogenomics (PGx) working groups.

**Results:**

HLA‐B*15:02 was most prevalent in South‐East Asia (5.6% ± 3.1), followed by South Asia (2.1% ± 1.5), North‐East Asia (0.6% ± 0.9), and West Asia (0.01% ± 0.05). This distribution was statistically significant (χ^2^ = 9712.5, df = 3, *p* < 0.001; *χ* ^2^ test). Country‐wise, prevalence was the highest in the Philippines (22%; 95% CI, 11.53‐35.96%), followed by Vietnam (13.5%; 95% CI, 8.77‐19.61%), Indonesia (11.9% ± 1.5), Malaysia (10% ± 4.3), Hong Kong (9.3% ± 0.2), Thailand (8.4% ± 0.1), Singapore (8.1% ± 3.7), China (5.4% ± 5.4), Taiwan (4.4% ± 0.7), Sri Lanka (2.5%, single study), India (2.1% ± 1.5) and South Korea (1.5% ± 0.9). PharmGKB clinical annotations identified four drugs (carbamazepine, oxcarbazepine, lamotrigine, phenytoin) with strong evidence (Level 1 A) for HLA‐B*15:02‐related SCARs, though the majority of the evidence for lamotrigine came from the Han Chinese Population. International PGx working groups require or recommend preemptive HLA‐B*15:02 genotyping for at least three drugs, i.e., carbamazepine, oxcarbazepine, and phenytoin.

**Conclusion:**

Considering the high prevalence of the HLA‐B*15:02 in the Southeast and South Asians, and its association with drug‐induced SCARs, pre‐emptive HLA‐B*15:02 testing may reduce SCARs substantially in this region.

## 1 Introduction

A set of cell surface proteins called the major histocompatibility complex (MHC) has a vital role in the activation of T‐cells, as the activation process necessitates the complementary engagement of T‐cell receptors with the antigenic peptides bound to the MHC molecules. The human leukocyte antigen (HLA) is the MHC present in humans, which is encoded by the *HLA* gene complex, situated on chromosome 6's short arm. Based on the locus and function of the coding gene, biochemistry, and tissue distribution, the *HLA* antigens are grouped under two clusters (i.e., class I and II). Three loci (i.e., classical *HLA‐A, HLA‐B*, and *HLA‐C* genes) encode for the molecules of *HLA* class‐I [1, 2]. The *HLA* genes exhibit a great degree of polymorphism resulting from different mutations, gene conversion, and meiotic recombination, accounting for a huge variability in the expression of *HLA* molecules [1, 2, 3, 4]. At present, more than 28,000 *HLA* class‐I alleles have been identified [5].

From a pharmacogenomics (PGx) perspective, *HLA* interactions have been associated with adverse drug reactions, resulting in hospitalization and even mortality, due to the drug‐induced hypersensitivity reactions (HSR) for several clinically important drugs. Due to the co‐dominant expression of *HLA*, the absence or presence of certain related *HLA* alleles dictates the susceptibility of drug‐induced HSR [1, 6, 7]. HLA‐B*15:02 is one such important allele that has been associated with the severe cutaneous adverse drug reactions (SCARs) of multiple extensively used drugs (e.g., carbamazepine, oxcarbazepine, phenytoin, etc.) [8, 9, 10, 11]. The SCARs are the delayed T‐cell induced HRS, such as Stevens‐Johnson syndrome/toxic epidermal necrolysis (SJS/TEN), acute generalized exanthematous pustulosis (AGEP), drug reaction with eosinophilic and systemic symptoms (DRESS) [1, 12]. Several international pharmacogenomics working groups, such as the Clinical Pharmacogenomics Implementation Consortium (CPIC), Canadian Pharmacogenomics Network for Drug Safety (CPNDS), and Dutch Pharmacogenetics Working Group (DPWG), have considered these genetic associations and proposed dosing, therapeutic, and testing guidelines intending to optimize the safety of the concerned drugs [13, 14, 15, 16].

However, the allele frequency of specific *HLA* alleles has been reported to exhibit significant variability across different ethnicities and populations [2]. Therefore, the implementation of the PGx‐based recommendations in clinical practice, importantly, the recommendation of preemptive testing heavily relies on the prevalence of the concerned allele in the population and the ethnicity in question [17]. The frequency of the HLA‐B*15:02 allele is significantly higher in Asian countries compared to the rest of the world, where the prevalence is extremely low [1, 16, 17]. One study estimated the total number of East and South Asian individuals carrying HLA‐B*15:02 to be 483.3 million, as compared to a total of only 3 million for non‐Asian individuals globally [17]. The genetic association for SCARs associated with drugs, such as carbamazepine, oxcarbazepine, lamotrigine, and phenytoin, is well documented and particularly of importance for the Asian populations, as evident in several studies. For example, the genetic association of HLA‐B*15:02 with the risk of lamotrigine‐induced SCARs is mainly established from the Asian (Chinese Han) population [18, 19, 20, 21, 22]. Considering the high allele frequency and variability among countries and populations and the potential implications for the safety of concerned drugs, a detailed study comparing the allele frequency in different Asian countries and regions is warranted to estimate the risk population to reinforce the necessity of genotype‐guided drug therapy. Such studies are scarce, and no prior study was identified that combined the allele frequency of the HLA B*15:02 in Asian countries, the risk of severe cutaneous adverse drug reactions (SCARs), with the PGx‐based dosing and testing recommendations.

Therefore, it was aimed to identify the prevalence of HLA‐B*15:02 among different Asian countries and regions by utilizing the allele frequency data from the Allele Frequency Net Database (AFND) [23] and correlate the prevalence information to the safety of certain drugs and the dosing, therapeutic, and testing recommendations, utilizing different international PGx‐based dosing guidelines.

## 2 Methods

### 2.1 Study Participants and Genetic Data

Information on allele frequency of HLA‐B*15:02 among different Asian populations was sourced from the Allele Frequency Net Database (AFND), a free‐to‐use repository of genetic frequency data of HLA, MHC Class I chain‐related genes, killer‐cell immunoglobulin‐like receptors (KIR), and a variety of cytokine gene polymorphisms in the world population. These data are curated from four major sources, namely, peer‐reviewed publications, individual laboratory submissions, population analyzed at the International HLA and Immunogenetics Workshops, and the short publication reports (SPR), the collaborative publication in the journal “Human Immunology”. As of the year 2020, AFND curated genetic data from over 10 million individuals across over 1600 global populations [24].

The allele frequency data of HLA‐B*15:02 for the Asian population from the AFND were identified and extracted by searching with locus B, selecting HLA‐B*15:02 allele and regions (South Asia, West Asia, North‐East Asia, and South‐East Asia) for the current study on March 9, 2025 [23]. All the sub‐populations with recorded allele frequency found with the search were included for the analysis. The AFND provided information about the allele frequency and % of individuals with the allele. However, due to the insufficient information on the latter, the present study only considered allele frequency for the analysis. We utilized the information of a total of 299,890 from 117 population groups originating from 16 different Asian countries and one special administrative region (Hong Kong) forming the four different Asian regions i.e., South Asia (India and Sri Lanka); South‐East Asia (China, Thailand, Hong Kong, Singapore, Malaysia, Taiwan, Vietnam, Indonesia and Philippines); West Asia (Iran, United Arab Emirates, Israel and Oman) and North‐East Asia (Japan and South Korea).

### 2.2 Linking the Allele Prevalence of HLA‐B*15:02 With the Safety of Clinically Important Drugs

To link the safety of several clinically used drugs to the HLA‐B*15:02 allele, we utilized the clinical annotation and PGx‐label information from international pharmacogenomics working groups, including PharmGKB, Health Canada Santé Canada (HCSC), Swissmedic, US Food and Drug Administration (FDA) approved drug label. All the information was extracted from the PharmGKB website [25, 26, 27]. Therapeutic and dosing guidelines for the identified drugs were sourced from different PGx‐based guidelines provided by the Clinical Pharmacogenomics Implementation Consortium (CPIC), Canadian Pharmacogenomics Network for Drug Safety (CPNDS), and the Dutch Pharmacogenetics Working Group (DPWG) [13, 14, 15, 16].

### 2.3 Statistical Analysis and Validation of Data Analysis

Descriptive statistics were utilized for the data analysis. The weight of each subpopulation was estimated from the sample size. Weighted means and standard deviations (SD) were then calculated using the following formulas [28],

$$\text{Weighted}\;\text{mean},\bar{x}w=\Sigma \left(w_{i}x_{i}\right)/\Sigma w_{i}$$

$$\text{Weighted}\;\text{Standard}\;\text{Deviation},\text{SD}=\surd \left[\Sigma w_{i}{\left(x_{i}-{\bar{x}}_{i}w\right)}^{2}/\Sigma w_{i}\right]$$

Where, wᵢ = weight of the sample, xᵢ = allele frequency of the subpopulation.

To investigate the discrepancies of the allele frequencies across different populations, the Chi‐square test was utilized, considering *p* < 0.05 as statistically significant. Both data analysis and graphical representation were performed using Microsoft Excel. For single study populations, we employed the Clopper‐Pearson exact binomial method to estimate the 95% Confidence Intervals (CI) for the allele frequencies using Epitools, a free to use online site for statistical analysis [29]. The Chi‐square test was performed using “Chi‐Square Calculator”, an online statistical analysis tool [30]. The analysis was performed independently by two researchers and amended accordingly if any anomaly was identified.

## 3 Results

### 3.1 Demography of the Participants and Prevalence of the HLA‐B*15:02 Allele

For the current analysis, the genetic information for the HLA‐B*15:02 allele among different Asian populations was collected from the AFND [23]. A total of 299,890 individuals from 17 counties conforming to four distinguished regions were included in the study. Sri Lanka and India, with a sample size (*n*) of 32,922 and 714, respectively, formed the South Asian region. Similarly, South‐East Asia featured China (*n* = 12,259), Thailand (*n* = 591), Singapore (*n* = 797), Malaysia (*n* = 1638), Taiwan (*n* = 49,661), Hong Kong (*n* = 12,056), Vietnam (*n* = 170), Indonesia (*n* = 479), Philippines (*n* = 50); West Asian region was comprised of Iran (*n* = 64), United Arab Emirates (*n* = 995), Israel (*n* = 155,674), Oman (*n* = 118) and the region of North‐East Asia was comprised of Japan (*n* = 19,993) and South Korea (*n* = 11,709). The detailed sub‐populations with sample size are outlined in Supporting Information Table S1.

The allele frequencies of HLA‐B*15:02 ranged among the different subpopulations studied. In India, it can be as high as 14.2% (Northeast UCBB), whereas the allele is absent in the West Coast Parsi population. Similar variations in the allele frequencies have been observed in Chinese subpopulations with frequencies ranging from 0% (Tibet Region Tibetan) to 35.8% (Yunnan Bulang). Figure 1a–e outlines the mean allele frequency of HLA‐B*15:02 in different sub‐populations of India, China, Malaysia, Singapore, and Taiwan, respectively.

Figure 1Prevalence of the HLA‐B*15:02 allele in different (a) Indian, (b) Chinese, (c) Malaysian, (d) Singaporean, and (e) Taiwanese sub‐populations.

**Figure:**
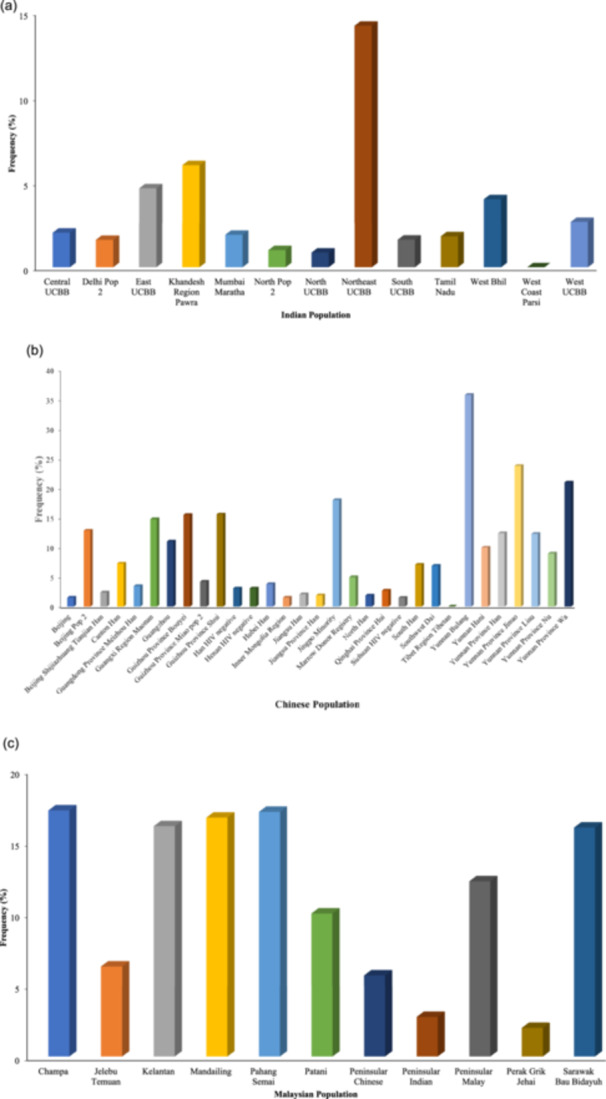

**Figure:**
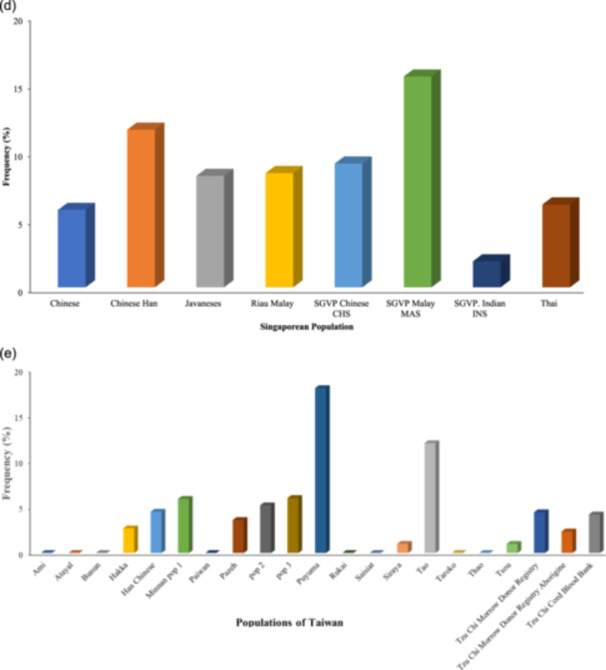

### 3.2 Mean Prevalence of the HLA‐B*15:02 Allele in Asian Countries and Regions

**Figure 2:**
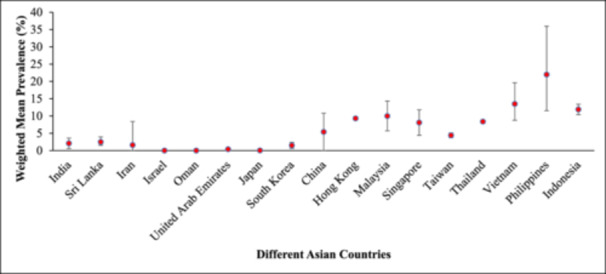
The prevalence of the HLA‐B*15:02 allele across different Asian countries. The error bars for the Philippines, Sri Lanka, Oman, Iran, and Vietnam represent the 95% confidence intervals, estimated from allele frequencies reported in a single source and thus should be interpreted with caution.

The weighted mean prevalence of the HLA‐B*15:02 allele in different Asian countries is shown in Figure 2. Across different Asian countries, the weighted mean prevalence of the HLA‐B*15:02 allele exhibited variation. The prevalence of the HLA‐B*15:02 allele was found highest in the Philippines (22%; 95% CI, 11.53–35.96%) followed by Vietnam (13.5%; 95% CI, 8.77–19.61%), Indonesia (11.9% ± 1.5), Malaysia (10% ± 4.3), Hong Kong (9.3% ± 0.2), Thailand (8.4% ± 0.1), Singapore (8.1% ± 3.7), China (5.4% ± 5.4), Taiwan (4.4% ± 0.7%), Sri Lanka (2.5%; 95% CI, 1.5–3.95%), India (2.1% ± 1.5), Iran (1.6%, %; 95% CI, 0–8.4%) and South Korea (1.5% ± 0.9), respectively. The weighted mean prevalence of the HLA‐B*15:02 alleles was found < 1% in the United Arab Emirates, Israel, and Japanese populations. It should also be noted that the 95% CI values were derived from single reports and is completely based on the statistical analysis and should be interpreted with caution.

**Figure 3:**
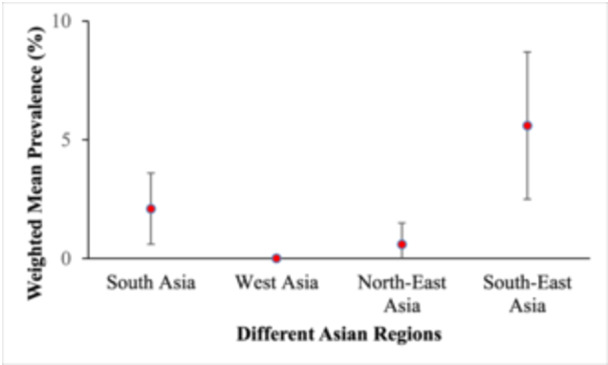
The prevalence of the HLA‐B*15:02 allele across different Asian regions.

Across different Asian regions, the prevalence of the HLA‐B*15:02 allele exhibited similar variation. The weighted mean prevalence of the HLA‐B*15:02 allele was highest in South‐East Asia (5.6% ± 3.1), followed by South Asia (2.1% ± 1.5), North‐East Asia (0.6% ± 0.9), and West Asia (0.01% ± 0.05), respectively. The prevalence of the HLA‐B*15:02 allele among different Asian regions (i.e., South‐East Asia vs. South Asia vs. North‐East Asia vs. West Asia) was statistically significant (χ^2^ = 9712.5, df = 3, *p* < 0.001; *χ* ^*2*^ test). Figure 3 illustrates the mean prevalence of the HLA‐B*15:02 allele in different Asian regions.

### 3.3 Linking HLA‐B*15:02 With Drug‐Induced SCARs

PharmGKB clinical annotations were utilized to correlate HLA‐B*15:02 with drug‐induced SCARs, which, based on a scoring system, assign a varied level of evidence from level 4 (unsupported) to level 1 A (high) to each drug, as detailed elsewhere [27]. This way, we identified for at least 6 drugs, i.e., carbamazepine, oxcarbazepine, lamotrigine, phenytoin, cotrimoxazole, dapsone, and one drug class, i.e., antiepileptics, in which the HLA‐B*15:02 was associated with drug‐induced SCARs [31]. Table 1 illustrates these associations. It's worth noting that the majority of the evidence for lamotrigine‐induced SCARs comes from the Han Chinese population and would benefit from further validation in other Asian populations.

**Table 1:** Association of HLA‐B*15:02 and a number of clinically important drugs.

Name of drug	Type of SCARs	Level of evidence
Carbamazepine	DRESS, TEN, SJS, MPE	1A
Oxcarbazepine	SJS	1A
Lamotrigine	TEN, SJS	1A
Phenytoin	DRESS, TEN, SJS	1A
Cotrimoxazole	TEN, SJS	2A
Dapsone	SJS	3
Oxcarbazepine	MPE	4

Abbreviations: DRESS = drug reaction with eosinophilia and systemic symptoms, MPE = maculopapular exanthema, SCARs = severe cutaneous adverse drug reactions, SJS = stevens‐johnson syndrome, TEN = toxic epidermal necrolysis. (Strong evidence primarily from Han Chinese populations; generalizability to other Asian subgroups requires further validation).

### 3.4 PGx Label Information of Certain Drugs Associated With HLA‐B*15:02

**Figure 4:**
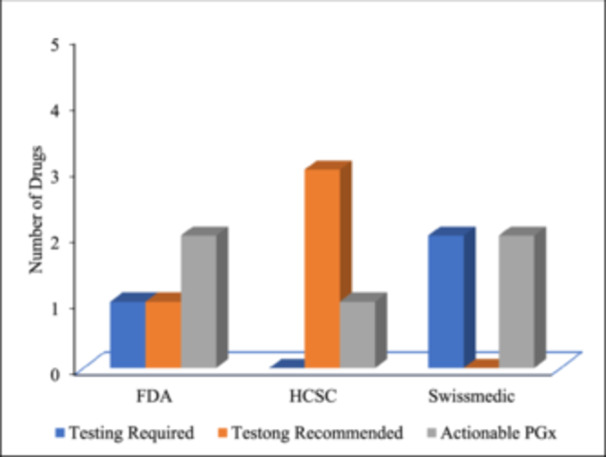
PGx label information from the FDA, HCSC, and Swissmedic for the drugs affected by the presence of the HLA‐B*15:02 (Here, FDA = Food and Drug Administration; HCSC = Health Canada Santé Canada; PGx = pharmacogenomics).

Due to the association with HLA‐B*15:02, the FDA, HCSC, and Swissmedic assigned at least 5 drugs with PGx labels, as shown in Figure 4. The FDA put a strong emphasis that genetic tests should be performed prior to using carbamazepine and recommended testing for oxcarbazepine and assigned two drugs, i.e., phenytoin and fosphenytoin, with “actionable PGx” label. Similar labeling of drugs was assigned by HCSC and Swissmedic, as detailed in Table 2.

**Table 2:** The pharmGKB drug labels for the HLA‐B*15:02.

Drugs	FDA	HCSC	Swissmedic
Carbamazepine	Testing Required	Testing Recommended	Testing Required
Oxcarbazepine	Testing Recommended	Testing Recommended	Testing Required
Phenytoin	Actionable PGx	Testing Recommended	Actionable PGx
Fosphenytoin	Actionable PGx	Actionable PGx	—
Pazopanib	—	—	Actionable PGx

Abbreviations: FDA = Food and Drug Administration, HCSC = Health Canada Santé Canada, PGx = Pharmacogenomics.

### 3.5 PGx‐Based Dosing and Therapeutic Recommendations

So far, different international PGx working groups have published therapeutic and dosing guidelines for at least five drugs. The CPIC has published the PGx‐based dosing guidelines for at least four drugs, i.e., carbamazepine, oxcarbazepine, phenytoin, and fosphenytoin on the basis of the HLA‐B*15:02 [14, 16]. The recommendations are classified as strong, moderate, optional, and no recommendation. The guidelines are detailed in Table 3A. The Canadian Pharmacogenomics Network for Drug Safety (CPNDS) provided similar guidelines for carbamazepine, and they graded the recommendations as Level A (strong), Level B (moderate), and Level C (optional) [13]. The guidelines are detailed in Table 3B. Dutch Pharmacogenetics Working Group (DPWG), on the other hand, provided PGx‐based therapeutic guidelines for carbamazepine, oxcarbazepine, phenytoin, and Lamotrigine [15]. The guidelines are detailed in Table 3C.

**Table 3A:** Clinical pharmacogenomics implementation consortium (CPIC) dosing and therapeutic guidelines based on HLA‐B*15:02 allele.

Drug [Reference]	Genotype	Dosing and therapeutic recommendations	Classification of recommendation
Oxcarbazepine [16]	HLA‐B*15:02 positive	Oxcarbazepine imposes an elevated risk of drug‐induced SJS/TEN and should br avoided in Oxcarbazepine‐naïve patients. Alternative aromatic anticonvulsants with weaker association with the HLA‐B*15:02 allele (e.g. eslicarbazepine, phenytoin, phenobarbital, fosphenytoin and lamotrigine) can be chosen, but with sufficient caution and careful consideration.	Strong
		Consider using oxcarbazepine in patients who have used it for over three months and exhibited no adverse reaction, as the drug‐induced adverse effects have a short latency period with continuous dosing for ~4–28 days and adherence to therapy, and the incidents are observed generally within 3 months.	Optional
Fosphenytoin, Phenytoin [14]	HLA‐B*15:02 positive	Avoid using these drugs, carbamazepine, and oxcarbazepine in Phenytoin‐naïve patients. Choose alternative drugs (eslicarbazepine, phenobarbital and lamotrigine) with a weaker association of SJS/TEN with HLA‐B*15:02.	Strong
		Consider using these drugs in patients who have used them for over three months and exhibited no adverse reaction, as the drug‐induced adverse effects have a short latency period with continuous dosing for ~4–28 days and adherence to therapy, and the incidents are observed generally within 3 months.	Optional
	HLA‐B*15:02 negative	Check for the predictive phenotype based on *CYP2C9* genetic polymorphism and reduce the dose accordingly for the IMs with an AS of 1 and the PMs.	Strong for PM and Moderate for IM with an AS of 1
Carbamazepine [16]	HLA‐B*15:02 negative	If the patient is HLA‐A*31:01 negative, then proceed using carbamazepine per standard dosing guidelines.	Strong
		Avoid using carbamazepine if the patient is HLA‐A*31:01 positive, carbamazepine naïve and alternative therapy is available.	Strong
		If no alternative therapy is available, use carbamazepine with continuous supervision and terminate therapy at the first sign of cADRs, in HLA‐A*31:01 positive carbamazepine naïve patients.	Optional
		Due to the latency period, if an HLA‐A*31:01 positive patient used carbamazepine continuously for more than 3 months without any events of cADRs, consider using carbamazepine with caution.	Optional
	HLA‐B*15:02 positive	As patients with any HLA‐A*31:01 genotype impose an elevated risk of toxicity, avoid using carbamazepine in carbamazepine naïve patients.	Strong
		As the toxicity reaction is generally exhibited within 3 months, in patients who used carbamazepine without any cADRs for over three months, the use of carbamazepine can be considered with caution.	Optional

Abbreviations: AS = activity score, cADR = cutaneous adverse reactions, IM = intermediate metabolizer, PM = poor metabolizer, SCARs = severe cutaneous adverse drug reactions, SJS = stevens‐johnson syndrome, TEN = toxic epidermal necrolysis.

**Table 3B:** Canadian pharmacogenomics network for drug safety (CPNDS) dosing and therapeutic guidelines based on HLA‐B*15:02 allele.

Drug [Reference]	Dosing and therapeutic recommendations	Grades of recommendation
Carbamazepine [13]	Alternative drugs should be considered as the first‐line therapy in HLA‐B*15:02 or HLA‐A*31:01 positive patients. Potential for cross‐reactivity of aromatic antiepileptic drugs with a similar structure (lamotrigine, phenobarbital, oxcarbazepine, phenytoin, primidone) should be considered while choosing alternative therapy.	Level A (Strong)
	In HLA‐B*15:02 and HLA‐A*31:01 negative patients, carbamazepine can be considered as the first‐line therapy, but on the basis of a negative result, the occurrence of hypersensitivity reactions cannot be ruled out.	Level A (Strong)

**Table 3C:** Dutch pharmacogenetics working group (DPWG) dosing and therapeutic guidelines based on the HLA‐B*15:02 allele.

Drug [Reference]	Genotype	Dosing and therapeutic recommendations
Phenytoin [15]	HLA‐B*15:02 positive	Carefully consider the benefits against the risk of SJS/TEN. If alternative therapy is possible, do not consider phenytoin. Due to carrying 10 10‐fold elevated risk of SJS/TEN, carbamazepine should not be considered as an alternative. Oxcarbazepine and lamotrigine carry comparable risk as phenytoin but however oxcarbazepine does not exhibit the most severe forms (i.e., TEN and SJS/TEN overlap). In unavoidable use fosphenytoin, advise patients to report skin rashes right away.
Carbamazepine [15]	HLA‐B*15:02 positive	Avoid carbamazepine. Lamotrigine, phenytoin and oxcarbazepine also pose similar risk of SJS/TEN but at much lower intensity than carbamazepine. With oxcarbazepine the most severe forms (i.e., SJS/TEN overlap and TEN) are not observed.
Oxcarbazepine [15]	HLA‐B*15:02 positive	Carefully determine the benefit versus the risk of SJS. If alternative therapy is possible, do not use oxcarbazepine. Because of the substantially elevated risk of SJS/TEN, carbamazepine should not be considered as an alternative. Though phenytoin and lamotrigine pose similar risks as oxcarbazepine, they may also not be considered as suitable alternatives. In the unavoidable case of oxcarbazepine use, advise the patients to report any skin rashes immediately.
Lamotrigine [15]	HLA‐B*15:02 positive	Carefully consider the benefit versus risk of SJS/TEN associated to the therapy. If alternative mediation is possible, avoid using lamotrigine. Due to the much higher risk associated, carbamazepine should not be considered as an alternative. Similar risks as lamotrigine are associated with phenytoin and oxcarbazepine, but however the use of oxcarbazepine does not exhibit the severe forms (i.e., SJS/TEN overlap and TEN). In unavoidable cases, advise the prompt reporting of any skin rashes.

### 3.6 PGx Testing Guidelines Considering HLA‐B*15:02

Based on the robust evidence of the incidences of carbamazepine induced SCARs associated with the HLA‐B*15:02 allele and a high prevalence of the concerned allele, CPIC and the FDA recommend genetic testing to screen for the HLA‐B*15:02 allele in the Asian population before the initiation of carbamazepine therapy [16, 32]. Similarly, DPWG recognizes the genotyping for HLA‐B*15:02 as ‘essential’ for carbamazepine therapy [15]. CPNDS strongly recommends (level A) genotyping prior to carbamazepine therapy in individuals originating from the population with high prevalence of the allele, e.g., Thai, Chinese, Indian, Filipino, Malay, Indonesian, and as optional (level C) for all patients [13]. CPIC also recommends genotyping of HLA‐B*15:02 for oxcarbazepine, phenytoin, and fosphenytoin therapies [14, 16]. For ensuring the safety of phenytoin, lamotrigine, and oxcarbazepine, DPWG recommends performing genotyping for HLA‐B*15:02 in the Asian population (other than Japanese) prior to or directly after the initiation of the therapy [15].

## 4 Discussion

A variability in the mean allele frequency of HLA‐B*15:02 across Asian regions was observed in this study. The highest prevalence of HLA‐B*15:02 was found in the South‐East Asian population (~6%), and South Asians (~2%) had the second highest prevalence. Country‐wise, Philippines (~22%), Vietnam (~14%), Indonesia (~12%), Malaysia (10%), Hong Kong (~9%), Thailand (~8%), Singapore (~8%), China (~5%), Taiwan (~4%), Sri Lanka (~3%) and India (~2%) had considerably high allele frequencies among Asian countries. However, from the varied SD values, it is evident that the frequency of the HLA‐B*15:02 allele also varies across different ethnic and sub‐population groups, necessitating a sub‐population‐focused approach as the risk of SCARs may be substantially higher or lower in certain sub‐populations. The observed prevalence data of the HLA‐B*15:02 allele was in line with the prevalence data reported in previous studies [1, 17].

The association of the HLA‐B*15:02 allele and drug‐induced SCARs for several drugs, namely, carbamazepine, oxcarbazepine, phenytoin, lamotrigine etc., has been well‐established in several meta‐analyses [8, 9, 18, 33, 34, 35, 36]. They found that these genetic associations are important for the Asian population, except for the Japanese, and may not bear much importance for the non‐Asian populations. This is particularly important for lamotrigine, as most of the evidence of the genetic association of SCARs and HLA‐B*15:02 has come from the Asian population, mainly Chinese Han. As we have observed a moderately high frequency of this allele among different Chinese populations, clinicians should focus on implementing PGx‐guided lamotrigine therapy for them.

Taking the solid evidence for the genetic associations into account, different international PGx working groups have provided therapeutic and dosing guidelines to implement the PGx guided therapy in clinical practice with an aim to optimize the safety. By compiling this approved information and other available evidence for genetic associations and comprehensively sorting it on the basis of its evidence level, the PharmGKB is turning into an essential PGx information tool. The PharmGKB also provided FDA, HCSC and Swissmedic label information for the drugs in concern due to the HLA‐B*15:02 allele. The FDA and Swissmedic assigned “testing required” and HCSC assigned “testing recommended” label for carbamazepine, emphasizing the need for proper genetic testing before initiating the therapy. Oxcarbazepine similarly was assigned to “testing recommended” label by the FDA and HCSC and “testing required” label by Swissmedic and for phenytoin HCSC recommended testing for HLA‐B*15:02. “Actionable PGx” label on the other hand discusses adjustment of the dose, recommendation for alternative therapy, contraindication and other therapeutic guidelines, though this label does not necessitate the pre‐emptive genotyping. “Actionable PGx” label due to HLA‐B*15:02 was assigned to phenytoin by the FDA and Swissmedic; to fospheytoin by the FDA and HCSC; to pazopanib by Swissmedic.

Given the severity of the adverse effects due to the HLA‐B*15:02 allele associated with certain drugs, dosing and therapeutic guidelines recommended by CPIC, CPNDS and DPWG may play an important role in the optimization of the safety of the concerned drugs in clinical practice. But it should also be noted that multiple other alleles may also be associated with the development of SCARs, for example, carbamazepine is affected by both HLA‐A*15:02 and HLA‐A*31:01; fosphenytoin and phenytoin are affected by *CYP2C9* and HLA‐B*15:02 [13, 14, 16]. So, following the testing recommendations, careful identification and evaluation of the combination of the genotypes is warranted. Development of an implementable polygenic risk score for these genetic associations may guide therapeutic decision‐making more efficiently.

The therapeutic guidelines encouraged the use of alternative medications (if available) or a reduced dose in the HLA‐B*15:02 positive patients. However, the chance of cross‐reactivity with the similarly structured drugs cannot be ruled out and should be carefully considered while choosing the alternative therapy. Similarly, a negative test result for the HLA‐B*15:02 allele does not eliminate the potential for the occurrence of SCARs entirely. For example, incidences of mild rashes in the HLA‐B*15:02 negative patients taking carbamazepine therapy have been reported, and this suggests that other genetic or non‐genetic factors may be involved in the development of carbamazepine/oxcarbazepine induced cutaneous adverse drug reactions [37]. It has also been reported that, alongside the HLA‐B*15:02 allele, other alleles, including HLA‐B*15:11, HLA B*15:08, and HLA‐B*15:21, may influence the safety of carbamazepine [1]. Validation of the role of these alleles in the development of SCARs with carbamazepine therapy and integration of that knowledge into PGx‐based guidelines should be considered.

Albeit the PGx working groups are continuously working to optimize the efficacy and safety of the drugs in clinical practice by utilizing the relevant PGx information, the application in real‐world settings remain limited. Specifically, the insufficient and incomplete correlation between the genetic variation and clinical evidence hinders the extensive application of PGx‐based precision medicines. Lack of proper cost‐effectiveness studies also contributes to the limited adoption of the PGx based therapy. So far, only in the Malaysian and Indonesian population have such cost‐effectiveness studies for the pre‐emptive HLA‐B*15:02 testing been conducted, and they found this screening to be cost‐effective [38, 39]. Replication of such studies with large and diverse population sets will provide a more comprehensive idea and advance precision medicine.

### 4.1 Limitations

Though the present study provides a good estimation of the mean allele frequency of the HLA‐B*15:02 for different Asian regions, data from only 16 of the Asian countries and one special administrative region (Hong Kong) were analyzed. A number of other Asian countries, e.g., Bangladesh, Nepal, Russia, Turkey, Uzbekistan, etc., thus remained unexplored. The review nature of the study and potential biases from the database source might also limit the generalizability of the findings.

### 4.2 Future Directions

As we observed a considerably high frequency of the HLA‐B*15:02 allele in South‐East Asia and South Asia, countries in this region should conduct thorough studies to identify the risk population and ethnic groups, and conduct large‐scale longitudinal studies and cost‐effectiveness analysis to optimize the safety of the concerned drugs in clinical practice.

## 5 Conclusion

A substantial proportion of Southeast and South Asian populations might be at a high risk of SCARs for at least 5 commonly prescribed drugs due to the presence of the HLA‐B*15:02 allele. It is a database‐dependent study, and further detailed sub‐population and ethnic analyses of the prevalence of the HLA‐B*15:02 allele and large‐scale clinical studies are imperative for the optimization of the safety associated with the concerned drugs and the adoption of PGx‐based precision medicine in clinical practice.

## Author Contributions

**Mohitosh Biswas:** conceptualization, investigation, writing – original draft, methodology, validation, visualization, formal analysis, data curation, supervision. **Murshadul Alam Murad:** investigation, writing – original draft, visualization, formal analysis, data curation. **Maliheh Ershadian:** writing – review and editing, methodology. **Chonlaphat Sukasem:** supervision, conceptualization, writing – review and editing, project administration, validation.

## Funding

The authors have nothing to report.

## Ethics Statement

The authors have nothing to report.

## Consent

The authors have nothing to report.

## Conflicts of Interest

The authors declare no conflicts of interest.

## Author Declaration

All authors have read and approved the final version of the manuscript. Dr. Chonlaphat Sukasem had full access to all of the data in this study and takes complete responsibility for the integrity of the data and the accuracy of the data analysis.

## Transparency Statement

Dr. Mohitosh Biswas affirms that this manuscript is an honest, accurate, and transparent account of the study being reported; that no important aspects of the study have been omitted; and that any discrepancies from the study as planned (and, if relevant, registered) have been explained.

## Supporting information


Supporting File

## Data Availability

The data that support the findings of this study are available in Allele Frequency Net Database at http://www.allelefrequencies.net. These data were derived from the following resources available in the public domain: –AFND, http://www.allelefrequencies.net. No datasets were generated. We utilized the AFND database, which is freely available (AFND, http://www.allelefrequencies.net).
