# Supplementary material for: Investigation of the Prevalence of the HLA‐B*15:02 Allele in the Asian Populations: A Comprehensive Analysis Through Using AFND
Source: Health Sci Rep. 2026 Jul 28;9(8):e72903. doi: 10.1002/hsr2.72903 (PMC13411286; doi:10.1002/hsr2.72903)
Supplement: Supplementary file 1 — Supporting File [file HSR2-9-e72903-s001.docx]

**Supplementary Table-S1.** The sample size of each subpopulation included in the study. All the information was sourced from **Allele Frequency Net Database (AFND) [1]**.

| **South Asia** | | |
| --- | --- | --- |
| **India** | | |
| **Population** | **Sample Size** | **Allele Frequency** |
| Central UCBB | 4204 | 2.0 |
| Delhi Pop 2 | 90 | 1.6 |
| East UCBB | 2403 | 4.6 |
| Khandesh Region Pawra | 50 | 6.0 |
| Mumbai Maratha | 91 | 1.9 |
| North Pop 2 | 72 | 1.0 |
| North UCBB | 5849 | 0.9 |
| Northeast UCBB | 296 | 14.2 |
| South UCBB | 11446 | 1.6 |
| Tamil Nadu | 2492 | 1.8 |
| West Bhil | 50 | 4.0 |
| West Coast Parsi | 50 | 0.0 |
| West UCBB | 5829 | 2.7 |
| **Sri Lanka** | | |
| **Population** | **Sample Size** | **Allele Frequency** |
| Colombo | 714 | 2.5 |
|  |  |  |
| **North-East Asia** | | |
| **Japan** | | |
| **Population** | **Sample Size** | **Allele Frequency** |
| Central | 371 | 0.1 |
| pop 16 | 18604 | 0.03 |
| pop 3 | 1018 | 0.1 |
| **South Korea** | | |
| **Population** | **Sample Size** | **Allele Frequency** |
| pop 10 | 4128 | 0.33 |
| pop 3 | 485 | 0.2 |
| pop 8 | 7096 | 2.2 |

| **South-East Asia** | | |
| --- | --- | --- |
| **China** | | |
| **Population** | **Sample Size** | **Allele Frequency** |
| Beijing | 67 | 1.5 |
| Beijing Pop 2 | 826 | 12.9 |
| Beijing Shijiazhuang Tianjian Han | 618 | 2.4 |
| Canton Han | 264 | 7.3 |
| Guangdong Province Meizhou Han | 100 | 3.5 |
| Guangxi Region Maonan | 108 | 14.8 |
| Guangzhou | 102 | 11.0 |
| Guizhou Province Bouyei | 109 | 15.5 |
| Guizhou Province Miao pop 2 | 85 | 4.2 |
| Guizhou Province Shui | 153 | 15.6 |
| Han HIV negative | 72 | 3.1 |
| Henan HIV negative | 16 | 3.1 |
| Hubei Han | 3732 | 3.8 |
| Inner Mongolia Region | 102 | 1.5 |
| Jiangsu Han | 3238 | 2.1 |
| Jiangsu Province Han | 334 | 1.9 |
| Jingpo Minority | 105 | 18.0 |
| Marrow Donor Registry | 600 | 5.0 |
| North Han | 105 | 1.9 |
| Qinghai Province Hui | 110 | 2.7 |
| Sichuan HIV negative | 34 | 1.5 |
| South Han | 284 | 7.1 |
| Southwest Dai | 124 | 6.9 |
| Tibet Region Tibetan | 158 | 0.0 |
| Yunnan Bulang | 116 | 35.8 |
| Yunnan Hani | 150 | 10.0 |
| Yunnan Province Han | 101 | 12.4 |
| Yunnan Province Jinuo | 109 | 23.8 |
| Yunnan Province Lisu | 111 | 12.3 |
| Yunnan Province Nu | 107 | 9.0 |
| Yunnan Province Wa | 119 | 21.0 |
| **Thailand** | | |
| **Population** | **Sample Size** | **Allele Frequency** |
| Thailand | 142 | 8.5 |
| Northeast pop 2 | 400 | 8.4 |
| pop 3 | 49 | 8.2 |
| **Singapore** | | |
| **Population** | **Sample Size** | **Allele Frequency** |
| Chinese | 149 | 5.7 |
| Chinese Han | 94 | 11.6 |
| Javaneses | 51 | 8.2 |
| Riau Malay | 132 | 8.4 |
| SGVP Chinese CHS | 96 | 9.1 |
| SGVP Malay MAS | 89 | 15.5 |
| SGVP. Indian INS | 86 | 1.9 |
| Thai | 100 | 6.1 |
| **Malaysia** | | |
| **Population** | **Sample Size** | **Allele Frequency** |
| Champa | 29 | 17.2 |
| Jelebu Temuan | 25 | 6.3 |
| Kelantan | 28 | 16.1 |
| Mandailing | 27 | 16.7 |
| Pahang Semai | 38 | 17.1 |
| Patani | 25 | 10 |
| Peninsular Chinese | 194 | 5.67 |
| Peninsular Indian | 271 | 2.77 |
| Peninsular Malay | 951 | 12.25 |
| Perak Grik Jehai | 25 | 2 |
| Sarawak Bau Bidayuh | 25 | 16 |
| **Hong Kong** | | |
| **Population** | **Sample Size** | **Allele Frequency** |
| Chinese | 569 | 10.2 |
| Chinese BMDR | 7595 | 9.38 |
| Chinese cord blood registry | 3892 | 9.12 |
| **Taiwan** | | |
| **Population** | **Sample Size** | **Allele Frequency** |
| Ami | 98 | 0.0 |
| Atayal | 106 | 0.0 |
| Bunun | 101 | 0.0 |
| Hakka | 55 | 2.7 |
| Han Chinese | 504 | 4.5 |
| Minnan pop 1 | 102 | 5.9 |
| Paiwan | 51 | 0.0 |
| Pazeh | 55 | 3.6 |
| pop 2 | 364 | 5.2 |
| pop 3 | 212 | 6.0 |
| Puyuma | 50 | 18.0 |
| Rukai | 50 | 0.0 |
| Saisiat | 51 | 0.0 |
| Siraya | 51 | 1.0 |
| Tao | 50 | 12.0 |
| Taroko | 55 | 0.0 |
| Thao | 30 | 0.0 |
| Tsou | 51 | 1.0 |
| Tzu Chi Morrow Donor Registry | 46682 | 4.4 |
| Tzu Chi Morrow Donor Registry Aborigine | 233 | 2.4 |
| Tzu Chi Cord Blood Bank | 710 | 4.2 |
| **Indonesia** | | |
| **Population** | **Sample Size** | **Allele Frequency** |
| Java pop 2 | 36 | 16.7 |
| Java Western | 236 | 12.2 |
| Sundanese and Javanese | 207 | 10.7 |
| **Philippines** | | |
| **Population** | **Sample Size** | **Allele Frequency** |
| philippines | 50 | 22 |
| **Vietnam** | | |
| **Population** | **Sample Size** | **Allele Frequency** |
| Hanoi Kinh pop 2 | 170 | 13.5 |

| **West Asia** | | |
| --- | --- | --- |
| **United Arab Emirates** | | |
| **Population** | **Sample Size** | **Allele Frequency** |
| Abu Dhabi | 52 | 0.96 |
| pop 1 | 570 | 0.4 |
| pop 2 | 373 | 0.6 |
| **Iran** | | |
| **Population** | **Sample Size** | **Allele Frequency** |
| Gorgan | 64 | 1.6 |
| **Israel** | | |
| **Population** | **Sample Size** | **Allele Frequency** |
| Georgia Jews | 4471 | 0 |
| Iran Jews | 8153 | 0 |
| Iraq Jews | 13270 | 0.01 |
| Kavkazi Jews | 2840 | 0.02 |
| Morocco Jews | 36718 | 0.03 |
| Poland Jews | 13871 | 0.01 |
| Tuinisia Jews | 9070 | 0.01 |
| USA Jews | 6058 | 0.01 |
| USSR Jews | 45681 | 0 |
| Yemen Jews | 15542 | 0 |
| **Oman** | | |
| **Population** | **Sample Size** | **Allele Frequency** |
| Oman | 118 | 0 |

**References:**

1. Allele Frequency Net Database. 2025 [cited 2025 Mar 09]; Available from: <http://www.allelefrequencies.net/>.
